# Severe hemolytic disease of the newborn caused by JK^b^ antibody: Two case reports and literature review

**DOI:** 10.1097/MD.0000000000034390

**Published:** 2023-07-28

**Authors:** Liang-Liang Jiang, Shao-Hua Bi, Jing Yu, Feng-Xia Zhao, Maggie Teng, Ru-Jeng Teng

**Affiliations:** a Pediatrics Neurology, Anhui Provincial Children’s Hospital, Hefei, China; b Division of Neonatology, Anhui Provincial Children’s Hospital, Hefei, China; c Division of Neonatology, Department of Pediatrics, Medical College of Wisconsin, Milwaukee, WI.

**Keywords:** blood exchange transfusion, hemolytic disease of the newborn, JK^b^ antibody, phototherapy

## Abstract

JK^b^ antibody rarely causes severe hemolytic disease in the newborn except in 1 case, required blood exchange transfusion but later died of intractable seizure and renal failure. Here we describe 2 cases of JK^b^-induced severe neonatal jaundice requiring blood exchange transfusion with good neurological outcome.

**Case presentation:**

Two female Chinese, ethnic Han, term infants with severe jaundice were transferred to us at the age of 5- and 4-day with a total bilirubin of 30.9 and 25.9 mg/dL while reticulocyte counts were 3.2% and 2.2%, respectively. Both infants were not the firstborn to their corresponding mothers. Direct and indirect Coombs’ tests were positive, and JK^b^ antibody titers were 1:64 (+) for both mothers. Phototherapy was immediately administered, and a blood exchange transfusion was performed within 5 hours of admission. Magnet resonance image showed no evidence of bilirubin-induced brain damage, and no abnormal neurological finding was detected at 6 months of life.

**Conclusion:**

JK^b^ antibody-induced hemolytic disease of the newborn usually leads to a benign course, but severe jaundice requiring blood exchange transfusion may occur. Our cases suggest good outcomes can be achieved in this minor blood group-induced hemolytic disease of the newborn if identified and managed early enough.

## 1. Introduction

Blood type incompatibility is a common cause of hemolytic disease in the newborn (HDN) but is rarely caused by minor blood groups. However, severe HDN secondary to minor blood group incompatibility that led to fatality has been reported, including Duffy, Kell, Kidd, etc. The Kidd (JK) glycoprotein is a urea transporter of the red blood cell.^[1]^ JK antibody can cause HDN, usually benign without complication.^[2]^ There was a total of 12 cases of JK^b^-HDN reported in English,^[2–13]^ including one required blood exchange transfusion (BET)^[3]^ and one with intrauterine fetal demise.^[4]^ The case reported by Kim died of renal failure and intractable seizure after a successful BET.^[3]^ Here, we present 2 severe JK^b^-HDN successfully managed by phototherapy and BET with normal neurodevelopmental outcomes.

### 1.1. Case 1

A 5-day-old term female infant of Han ethnicity was born to a G_2_P_2_ mother at 40 weeks of gestation, vaginally, with a birthweight of 3500 g and Apgar scores of 10 and 10. Her weight was 3520 g upon admission. She was fed with both breast milk and formula. The first bowel movement happened at 4 hours of birth. No hematoma was noted, and no family history of neonatal jaundice. The mother was never exposed to blood transfusion and denied any autoimmune disease. There was no ABO or Rh incompatibility. Her 21-month-old sister was in good health. The admission hemogram showed Hb 126 g/L, Hct 35%, and a reticulocyte count of 2.2%. A blood smear showed anisocytosis, microspherocytosis, and elliptocytosis. The total and direct bilirubin levels were 309 and 19 mg/L, respectively. Coombs’ tests were positive. Anti-JK^b^ antibody (Sanquin Reagents B.V., Amsterdam, Netherlands) was 1:64 (+) in maternal blood. The level of G-6-P-D was normal, and no evidence of urinary tract infection. No intracranial hemorrhage was found by cranial ultrasound. Thyroid function was normal. Intensive double phototherapy was initiated immediately after admission. Type O (+) JK^b^(−) packed red blood cells and type AB (+) plasma were mixed for BET performed 5 hours after admission. The total and direct bilirubin levels were 13.9 and 1.0 mg/dL immediately after BET with Hb 129 g/L, Hct 39%, and a reticulocyte count of 1.7%. Phototherapy was continued for another day after the BET. On the 3rd day of admission, the brain MRI showed no obvious abnormality, and she passed the hearing screening. She was discharged at 11 days of life with Hb 122 g/L, Hct 37%, and a reticulocyte count of 1.5%. Neurological examination at 6-month-old showed no abnormal finding.

### 1.2. Case 2

A 4-day-old term female infant of Han ethnicity was born G3P2 mother at 37 weeks of gestation via Cesarean section. The birth weight was 3200 g. Her admitting weight was 3150 g. Apgar scores were 8 and 10, respectively. She was visually jaundiced at the 36th hour of birth. She had normal activity without a high-pitch cry. She was fed with breast milk and formula. The first bowel movement was at 10 hours of life. No hematoma was seen, and no family history of neonatal jaundice. The mother was never exposed to blood transfusion and denied autoimmune disease but did receive instrumental abortion for her second pregnancy. No evidence of ABO or Rh incompatibility. Her older brother, 4 years old, was in good health without neonatal jaundice. Upon admission, the hemogram showed Hb 139 g/L, Hct 36%, and a reticulocyte count of 3.2%. A blood smear showed anisocytosis, elliptocytosis, fragmented red blood cells, and polychromasia. The total and direct bilirubin levels were 259 and 9 mg/L, respectively. Coombs tests were positive. Anti-JK^b^ was 1:64(+) in maternal blood. The level of G-6-P-D and urinalysis were normal with sterile urine. No intracranial hemorrhage was found by cranial ultrasound. Thyroid function was normal. The patient was given double phototherapy immediately after admission. BET was performed 4 hours after admission with a mixture of Type O(+) JK^b^(−) packed red blood cells and type AB (+) plasma. The immediate post-exchange transfusion total and direct bilirubin levels were 106 and 6 mg/L, respectively. Hemogram after exchange transfusion showed Hb 137 g/L, Hct 39%, and a reticulocyte count of 1.5%. On the 7th day of birth, her brain MRI showed no abnormality, and she passed the hearing screening. She was discharged at 9 days old with Hb 133 g/L, Hct 38%, and a reticulocyte count of 1.4%. Neurological examination at 6-month-old showed no abnormal finding.

## 2. Discussion

ABO and Rh-incompatibility are the most common cause of HDN and should always be considered in neonates with severe jaundice. Rh-HDN is more severe than ABO-HDN before the introduction of Rhogam.^[14]^ HDN due to other minor blood groups were subsequently identified, such as Kell, Duffy, Kidd (JK), and MN antigens.^[15]^ Minor blood group HDNs need a sensitized mother, so they are more commonly seen in non-first-born infants. With the recent abolition of the Chinese Population Policy, we have started to experience more HDN caused by minor blood group incompatibility.^[14]^

Kidd blood group is an antigen in human erythrocytes, which consists of 2 specific genes (JK^a^ or JK^b^). Kidd antigen is a 43 kDa urea transporter on the erythrocyte membrane, and its deletion is compatible with life with limited urea concentrating ability of the affected kidneys^.[1]^ Among Asians, JK^a^ and JK^b^ account for 49%, similar to other ethnic groups.^[1]^ The reported gene frequency in Chinese Han population is 48.4% for JK^a^ and 51.6% for JK^b^.^[16]^ JK^a^ and JK^b^ have very weak immunity and rarely elicit an immune reaction. There are 2 types of antibodies produced against Kidd antigen, IgG and IgM, but only the IgG isotype will cause HDN. The mechanism by which high titers of JK^a^ and anti JK^b^ antibodies are generated remains unclear.

In 1953, Plaut et al^[17]^ described the JK^b^ antibody. Most reported JK^b^ incompatibility occurred in adults after repeated transfusion.^[18]^ Allen was the first to identify an antibody in the maternal blood against the JK^a^ of a newborn with HDN. The blood group, Kidd, was hence named after that mother’s maiden name.^[19]^ Kornstad and Halvorsen^[5]^ reported the first case of JK^b^-HDN in 1958. Presently only 12 cases of JK^b^-HDN have been reported in English literature (Table 1).^[2–13]^ Most of the JK^b^-HDN (12/14, 85.7%) had a very mild course and required no more than phototherapy. There were 2 fatal cases (14.3%); one died of renal failure and intractable convulsions despite BET and phototherapy,^[3]^ while the other ended with intrauterine fetal demise at 25 weeks of gestation.^[4]^ Two patients (14.3%) received simple blood transfusions, and 3 (21.4%) received blood exchange transfusions. Our patients received BET according to the AAP guidelines. Brain MRI and BAEP were normal at the time of discharge. Neurological examination in both cases was normal after 6 months.

**Table 1:** Summary of the clinical and laboratory data from the published cases of hemolytic disease of newborns due to anti-Jk^b^.

Reference	2	3	4	11	12	13	14	15	16	17	18	19	*#1*	*#2*
GA (wk)	Term	37	***25***	41	Term	37	Term	Term	38	Term	37	36	Term	Term
Onset (h)	48	NM	Birth	4	NM	2	NM	NM	Birth	30	10	NM	48	36
Peak T-bil (d)	2	4	NM	2	2	1	2	1	2	2	2	3	5	4
Peak T-bil (mg/L)	86	461	NM	103	90	49	84	80	168	144	181	146	309	259
Hb (g/L)	123	114	NM	120	148	212	NM	170	124	188	NM	98	126	139
Ht (%)	37.1	36.8	NM	NM	NM	61	60.5	NM	37.4	NM	50	NM	35	36
Ret (%)	NM	14.9	NM	6.4	4.6	NM	NM	NM	6.7	6.0	10.4	>10	2.2	3.19
Treatment	None	PT/**ETF**	TF	None	None	None	None	None	PT/TF	NM	PT	PT/TF	PT/**ETF**	PT/**ETF**
Outcome	Good	**Fatal**	**Fatal**	Good	Good	Good	Good	Good	Good	Good	Good	Good	Good	Good
G-P	3-3	4-2	4-3	5-4	2-1	3-3	5-4	NM	3-3	4-3	7-1	NM	2-2	3-2
HDN siblings	None	1st/2nd child	None	None	None	NM	None	NM	2nd child	None	1st (?)	NM	None	None

ETF = exchange transfusion., GA = gestational age, G-P = gravida-para, Ht = hematocrit, NM = not mentioned, PT = phototherapy, Ref = reference, Ret = reticulocyte count, TF = transfusion, Tx = treatment.

Phototherapy and BET are the standard measures for severe HDN. To establish the true cause of HDN, when ABO or Rh incompatibilities can be excluded, we need to consider other minor blood group antibodies. Due to their low antigenicity, most minor blood group HDNs occur either not as the first child, or the mothers have previous exposure to transfusion or abortion. Antenatal ultrasound can detect fetal anemia or hydrops caused by severe hemolysis, and intrauterine transfusion may be offered.^[20]^ An intimate collaboration between the perinatologist and neonatologist is needed to care for such a situation. Although most JK^a^/JK^b^ HDN have a benign clinical course, the potentially fatal outcome cannot be ignored. About 50% of pregnant Chinese women are JK^b^ positive, which gives them roughly 25% chance of having JK^b^ incompatibility. Luckily, severe JK^b^-HDN requiring aggressive management is extremely rare. This implies that universal detection of JK^a^/JK^b^ antibodies, or other minor blood group antibodies, will be clinically unnecessary. Universal screening of neonatal jaundice, either by blood test or transcutaneous method, will be very important to prevent bilirubin induce brain damage. Severe bilirubin induce brain damage such as kernicterus has a devastating consequence for the victims, their families, and society. We want to advocate a national policy to implement measures to prevent kernicterus which is especially important after the discontinuation of our national population policy since we do expect severe HDN due to minor blood groups will become more frequent.

Fortunately, with intensive phototherapy followed by successfully performed BET we obtained good outcomes for 2 severe JK^b^-HDN. We want to share our experience with our peers to draw attention to the adequate management of severe HDN regardless of the etiology.

## Acknowledgments

We want to express our appreciation towards all the nursing staff, medical technicians, and medical informatics of the Hospital and Regional Blood Center. This report could not happen without their dedication to the care of our patients.

## Author contributions

**Data curation:** Feng-Xia Zhao.

**Writing – original draft:** Liang-Liang Jiang.

**Writing – review & editing:** Shao-Hua Bi, Jing Yu, Maggie Teng, Ru-Jeng Teng.
